# Clinical Remarks upon Surgical Cases in the Buffalo General Hospital—Syphilitic Necrosis of the Skull—Trephining

**Published:** 1866

**Authors:** J. F. Miner


BUFFALO

### Medical and Surgical Journal.

VOL. V. MARCH AND APRIL, 1866. Nos. 8 and 9.

ART. I—*Clinical Remarks upon Surgical Cases in the Buffalo General Hospital — Syphilitic Necrosis of the Skull — Trephining.* By J. F. Miner, M. D.

*Gentlemen:*—We are about to present before you a case of syphilitic necrosis of the cranial bones, and to attempt the removal of whatever may be found ulcerated or dead. The case will illustrate how fearful is the penalty of transgression, and show that there is no known method of preventing in some instances the terrible consequences of a disease which pervades every fluid, and every solid in the whole system, and which springs up after years of repose to complete a mission for evil which has no bounds or estimate. It is not classed as a malignant disease; medicine has greater influence for positive cure in syphilitic diseases than in almost any other; we almost boast of a specific, but we are often painfully reminded of a fact which we sometimes seem to forget, "that such boasting is vain." Mercury, properly administered, will, in most constitutions, control the secondary effects of syphilis. It is also sometimes useful in other stages of the disease, but it is not a specific, and will not wholly eradicate it from the system at any time. It is capable of good, it is also potent for evil, and as it generally has been, and still is, prescribed, quite as often works mischief as benefit. In the primary lesion, it may generally be omitted; it will not prevent constitutional infection, and possibly by uniting with the poison of syphilis, may produce a mercuro syphilitic disease more stubborn and incurable than if only one poison were to be eradicated. The primary sore may be healed without mercury, by local treatment; the secondary disease is greatly controlled by it, not wholly eradicated; while the later manifestations which we call tertiary symptoms, are more influenced by iodine in its various combinations. The patient which is presented this morning, has passed through the hands of several physicians distinguished for heroic medication, and probably has not lacked for a supply of the "all healing panacea of syphilis;" however, we may not open this closed volume. Since his admission to the surgical ward of this hospital he has taken no medicine, and has not appeared to suffer from this neglect. He suffers from most profuse discharge of pus from the sinuses you observe upon the forehead, and a probe detects nenuded bone, that is, bone denuded of its periosteal covering; by firm pressure it appears that a portion of the os frontis is dead, and separating from the living bone beneath, that there is exfoliation of the outer table of bone. With the view of determining the condition more exactly, and of removing any dead bone which may be found, we make incisions down upon the skull and expose to view the dead, darkened, eburnated bone of the cranium. It is now seen to be dead, but it is not so much separated as appeared, and we will cut out a central portion with trephine, to ascertain the extent of the necrosis; when perhaps the fragments may be easily removed. It will now be seen that not only the outer table as we supposed is diseased, but the entire bone, including both tables. We have then trephined the skull, and you can now see that the membranes lining it are diseased and separated, and that large quantities of pus lay under the bone, relieved from pressing upon the brain by openings which had formed by partial separation of the dead from the living and healthy bone. By this operation we hope the sooner to get rid of the dead portion, and thus to lessen the drain upon the system caused by the profuse suppuration.

You may inquire if I think this disease is due to the effects of mercury administered for the cure of syphilis? and such question is worthy of careful consideration. Mercury, in some constitutions, improperly and extravagantly administered, is believed upon good grounds to have induced death of bone. Inflammation of the periosteum terminating in suppuration or in separation from its attachments may cause death of bone, by arresting the circulation—cutting off the supply. Mercury has been supposed to be capable of producing such results, and it is probable that this belief is sustained by abundant proof. Syphilis is constantly observed to produce nodes—inflammation of the periosteum; which inflammatory action may be very active and acute, and cause arrest in the circulation of the bone to an extent inconsistent with life. Two causes then appear capable of producing this result, both operating in the same way, causing death of bone by arrest of circulation and consequent loss of nutrition, or by inducing inflammation of bone independently of any affection of external parts. Will syphilis produce these results in the bones and their periosteal coverings without the aid of mercury, or will such results follow in any case where no mercury has been administered? Does it require a combination of mercury with the poison of syphilis to produce these melancholy results?

The idea which is often advanced that mercury is necessary in the production of such results is wholly unsustained. That even its judicious employment in the treatment of syphilis may favor such disease has been been believed, but there can be no doubt that properly administered it is curative in its action, and often necessary in conjunction with iodide of potassium for the most permanent removal of the disease attainable.

Inflammation of the cranial bones, resulting in caries or necrosis is said to generally commence in the external table, but sometimes as in the case before you, it either commences in the internal table or extends from without inwards, in which event the products must either find exit through perforations or soon cause compression of the brain, or inflammation and disorganization of the cerebral substance. The frontal bone is much more frequently than other cranial bones attacked; all of the bones of the skeleton are exposed to such disease.

When caries or necrosis occurs, we should never fail to remove the diseased portion as soon as it can be separated from the sound parts. "Caries engenders caries," and when the tissue of bone has been destroyed, it can never be recovered by any means whatever; it becomes a foreign body, and by maintaining and extending suppuration produces depressing and injurious effects which might even result in death. We hope in this case to be able to remove all dead and separated portions of bone and to relieve the patient of a drain which has reduced a naturally healthy and vigorous patient to a condition of alarming debility and depression.*

* At this present time, two months after the operation, the suppuration has nearly ceased; all diseased bone is removed and the wound nearly healed; the dura mater, which at the time of operation was found greatly diseased, thickened and ulcerated, has been slower in recovering itself than the bony structure, and the present slight secretion of pus appears to be wholly furnished by that membrane. The results of the operation thus far have been in the highest degree satisfactory

